# Meta-Analysis of Quantitative Traits Loci (QTL) Identified in Drought Response in Rice (*Oryza sativa* L.)

**DOI:** 10.3390/plants10040716

**Published:** 2021-04-07

**Authors:** Nurhanis Selamat, Kalaivani K. Nadarajah

**Affiliations:** Department of Biological Sciences and Biotechnology, Faculty of Science and Technology, University Kebangsaan Malaysia, Bangi 43000 UKM, Malaysia; A162669@siswa.ukm.edu.my

**Keywords:** rice (*Oryza sativa* L.), drought, QTLs, consensus map, meta-QTL, drought related traits, drought related genes

## Abstract

Rice is an important grain that is the staple food for most of the world’s population. Drought is one of the major stresses that negatively affects rice yield. The nature of drought tolerance in rice is complex as it is determined by various components and has low heritability. Therefore, to ensure success in breeding programs for drought tolerant rice, QTLs (quantitative trait loci) of interest must be stable in a variety of plant genotypes and environments. This study identified stable QTLs in rice chromosomes in a variety of backgrounds and environments and conducted a meta-QTL analysis of stable QTLs that have been reported by previous research for use in breeding programs. A total of 653 QTLs for drought tolerance in rice from 27 genetic maps were recorded for analysis. The QTLs recorded were related to 13 traits in rice that respond to drought. Through the use of BioMercartor V4.2, a consensus map containing QTLs and molecular markers were generated using 27 genetic maps that were extracted from the previous 20 studies and meta-QTL analysis was conducted on the consensus map. A total of 70 MQTLs were identified and a total of 453 QTLs were mapped into the meta-QTL areas. Five meta-QTLs from chromosome 1 (MQTL 1.5 and MQTL 1.6), chromosome 2 (MQTL2.1 and MQTL 2.2) and chromosome 3 (MQTL 3.1) were selected for functional annotation as these regions have high number of QTLs and include many traits in rice that respond to drought. A number of genes in MQTL1.5 (268 genes), MQTL1.6 (640 genes), MQTL 2.1 (319 genes), MQTL 2.2 (19 genes) and MQTL 3.1 (787 genes) were annotated through Blast2GO. Few major proteins that respond to drought stress were identified in the meta-QTL areas which are Abscisic Acid-Insensitive Protein 5 (ABI5), the G-box binding factor 4 (GBF4), protein kinase PINOID (PID), histidine kinase 2 (AHK2), protein related to autophagy 18A (ATG18A), mitochondrial transcription termination factor (MTERF), aquaporin PIP 1-2, protein detoxification 48 (DTX48) and inositol-tetrakisphosphate 1-kinase 2 (ITPK2). These proteins are regulatory proteins involved in the regulation of signal transduction and gene expression that respond to drought stress. The meta-QTLs derived from this study and the genes that have been identified can be used effectively in molecular breeding and in genetic engineering for drought resistance/tolerance in rice.

## 1. Introduction

Rice (*Oryza sativa* L.) is one of the most important cereals in the world. It is a major food source for more than half of the world’s population. More than 90% of rice is grown and eaten by Asians, who make up 60% of the world’s population. Rice cultivation is also a major source of income for the rural population [1]. The Southeast Asian Region (SEA) has become the world’s economic center of rice [2]. In 2016, the Asian region produced 453.2 million tons of rice, representing 90% of the world’s rice production [3]. The demand for rice is expected to increase in line with the ever-increasing population rate. Therefore, Asian countries are always concerned about ways to obtain an adequate supply of rice in line with the growing demand [3]. Rice has the potential to produce a yield of 10 tons per hectare. However, the average rice yield produced by local farmers is only between 4–5 tonnes per hectare [4]. The high gap in the rice yield is due to various environmental pressures. Therefore, in order to achieve food security status in rice production by 2050, the development of high yielding rice varieties with tolerance to both biotic and abiotic stresses is crucial [5].

Environmental stress is divided into two factors, namely biotic and abiotic stresses. Biotic stresses are caused by organisms, such as fungi, bacteria, viruses, parasites, weeds, and insects [6]. This will cause diseases and pest predation that will result in loss of yield. Rice blast, sheath blight and bacterial leaf blight are the major rice diseases reported in rice. Kumar et al. mapped the relationship between defense related and resistance genes to build a defense model for diseases in rice [7]. The major R-genes found in specific QTLs can be utilized in developing resistant cultivars [8]. Further, a meta-analysis for the quantitative traits loci combining these three diseases identified resistance QTLs for use in both molecular breeding and genetical engineering [9]. 

Abiotic stress refers to negative effect on plants caused by certain environments [10] such as drought, high salinity, flooding, and other extreme environments. Abiotic stress is one of the main factors causing decreased crop productivity worldwide, thus jeopardizing food safety status [11]. Among these pressures, drought is a major stress that causes a decline in rice agricultural production worldwide. Singhal et al. [12] estimated that more than 50% of the world’s agricultural land will be affected by drought by 2050. Compared to other grain crops such as corn and wheat, rice is more sensitive to water content in the soil as rice is a semi-aquatic plant, which has been planted in flood irrigated conditions where the soil matrix potential is zero [13]. In 2016, rice cultivation could only be carried out in 30% of the total 11,500 hectares of rice fields in the Northern Regions of Malaysia due to drought [14]. Drought can affect plants in a variety of ways such as growth, crop yield, membrane integrity, pigment content, osmotic adaptation, and photosynthetic activity [15]. Thus, drought is a constant threat to food security and rice production worldwide.

Drought tolerance is defined as plant tolerance towards a state below the minimum level of moisture content in the cytoplasm i.e., when water content forms approximately 23% fresh tissue and 0.3 g dry tissue [16]. The nature of drought tolerance in rice is complex and is determined by various characteristics. Many genes that have high environmental interactions as well as low heritability determine these characteristics. Therefore, it is a feature that is difficult to study [17]. Most modern rice is sensitive to drought, and its worst effects are seen during the reproductive stage. Conventional breeding methods used to increase rice tolerance towards drought is slow and limited due to the quantitative nature of drought resistance [18]. Since drought is a major problem in rice, the identification of genomic areas that affect the yield response and its components will help improve our understanding on the mechanisms involved in drought resistance and assist in breeding for drought resistance or tolerance [19].

Through the resulting development in genome technology such as molecular markers and genetic engineering, studies to identify genes or quantitative trait loci (QTL) related to drought resistance in rice have been extensively employed to increase molecular understanding of drought resistance [20]. QTL analysis is important in agriculture as it provides knowledge of the desired QTL location and can be used in breeding programs [21]. Molecular markers for specific QTLs can help to improve the appropriate recombinant selection of the best plant varieties and accelerate the breeding program [22]. Studies of QTLs related to drought resistance are needed to identify individual genes in order to aid in the improvement of selected breeding especially when the nature of drought tolerance has low inheritance. Currently, many QTLs for drought tolerance in rice have been identified. Most of these QTLs in rice have been identified based on various important characteristics such as rice yield, root and root response, osmotic adaptation, hormonal response, photosynthesis and overall plant response to drought tolerance [16,23,24]. Thus, this study is important in identifying QTLs in rice (*Oryza sativa* L.) for tolerance towards drought that are stable in all environments and genotypes for effective use in MAS. The markers linked to these meta-QTLs can be utilized in the screening of resistant progenies in MAS and thus shorten the time required to generate new varieties. Key genes identified within these MQTLs may be useful in genetical engineering of rice.

## 2. Results and Discussion

### 2.1. Collection of QTL Data Related to Drought Tolerance in Rice from Past Studies

A total of 20 studies related to drought tolerance in rice was collected and selected for further analysis. The reports of these studies cover various rice varieties, population size, crossing methods and environmental conditions. Table 1 shows the information of the articles selected for use in this study. From the 20 articles, 27 QTL genetic maps were extracted and integrated into one QTL genetic map related to drought tolerance in rice. A total of 653 QTLs for drought tolerance in rice were recorded. Collected QTLs include QTLs related to properties that respond to drought stress. A total of 13 traits have been obtained from this bibliographic study. The QTLs for traits assessed in this study only take into account the QTLs that were expressed in drought conditions. There are few previous studies that used both drought and control conditions. The QTLs that were expressed in control conditions had different positions in the chromosomes, different LOD and phenotypic variation although these QTLs were related to the same traits. This shows that, rice will express different QTLs according to the environmental conditions. Since the current study is to find meta-QTL related to drought tolerance in rice, only the QTLs expressed in drought condition were taken into account.

Various molecular markers such as Short Simple Sequences (SSR) markers, Restriction Fragment Length Polymorphism (RFLP), Amplified Fragment Length Polymorphism (AFLP), Randomly Amplified Polymorphic DNA (RAPD) and Single Nucleotide Polymorphism (SNP) linked to these QTLs were also collated. The information collated enabled us to identify QTL “hotspots” for drought resistance in rice as well as stable QTLs in various varieties, crossing methods and environment. The “hotspots” are areas on the rice chromosome that are frequently reported in several genetic and environmental backgrounds. Due to the stability of these “hotspot”, they are useful in breeding for resistance.

Through the collection of QTL data from previous studies, 13 traits of rice related to rice response to drought stress such as grain yield (GY), drought score (DS), plant height (PH), biomass (Bio), roots (Rt), shoots (Sh), leaves (Lf), water (Wat), temperature (Temp), flowers (FL), recovery score (RS), stem (St) and seeds (Sd) were identified. Out of the 653 QTLs associated with rice tolerance to drought, 169 QTLs were related to tree root traits. This is relevant because the roots are organs that function to absorb water and nutrients from the soil and are directly related to the physiology of the tree. The roots of the tree is the first organ exposed to drought stress pressure as this is where the lack of water stress is applied [25]. Therefore, in the event of a water shortage due to drought, the roots of the plant are severely affected. This makes the root widely studied in relation to drought stress, where the morphological and physiological characteristics of the roots play an important role in determining the growth of shoots as well as the yield of the plants [25]. QTLs related to the flowering traits (118) are the second highest recorded. The flowers are the reproductive organs that when affected by drought, will indirectly affect the yield of paddy drastically [26].

**Figure 1:**
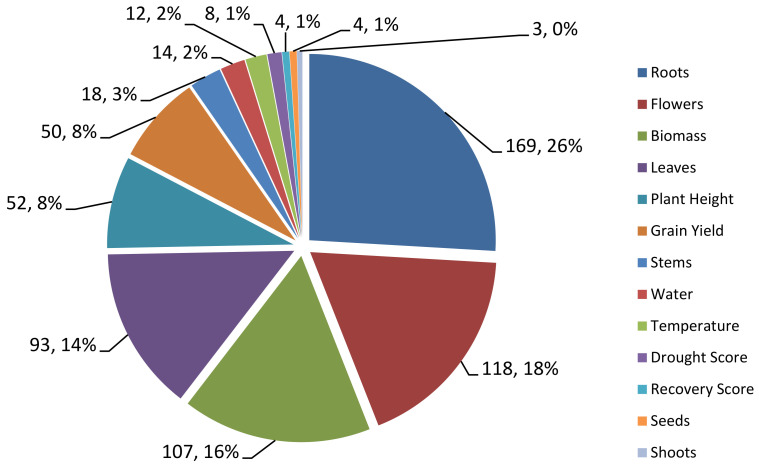
Pie chart of QTL data distribution based on rice traits. Rice traits related to roots have the highest numbers of QTL while traits related to seeds and shoots have the lowest percentage of QTLs.

Next, the QTL related to the biomass traits recorded 107 QTL. The changes in the roots and leaves will affect the biomass of the plant. Paddy biomass is reduced in terms of dry weight or wet weight due to drought stress which indirectly affects crop yield [27]. QTLs related to the leaves trait were also relatively large with 93 QTLs. As reported by Upadhyaya & Panda [27], the size and number of leaves on each rice plant decreases when there is water pressure from the soil. The report also stated that high drought stress resulted in the overall leaf area reduction in Nagina 22 (N22). This is in line with the relatively large number of QTLs recorded for the leave trait as this organ is among those affected when faced with drought stress as seen in leaf rolling and wilting. QTLs related to other traits such as grain yield, drought score, plant height, water, temperature, and stem contributed to anything between 12 to 52 QTLs in this bibliographic study. The least QTLs obtained are QTLs related to tree shoots which is only 3 QTLs and this is followed by tree seed and recovery score with 4 QTLs. Although these traits were also affected when faced with drought stress, less QTLs were reported as they are not the primary organs or processes affected by drought in rice or plants [27]. The overall distribution of the QTLs based on these 13 traits is shown in Figure 1.

### 2.2. Generation of Consensus Map and Projection of QTLs

BioMercator V4.2 was used for QTL mapping and incorporation of molecular markers related to rice tolerance to drought. A consensus map was generated from 27 genetic maps that had been reported from previous studies. This consensus map was generated to determine the QTL co-localization regions on each rice chromosome. 

**Figure 2:**
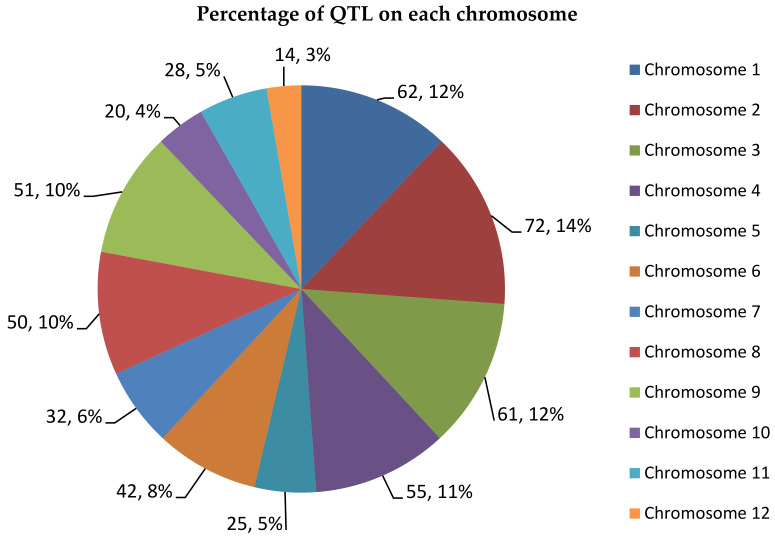
QTL distribution in each rice chromosome. The pie chart provides the number of QTLs in each chromosome and the percentage of these QTLs based on the total number of QTLs in the 12 chromosomes.

**Figure 3:**
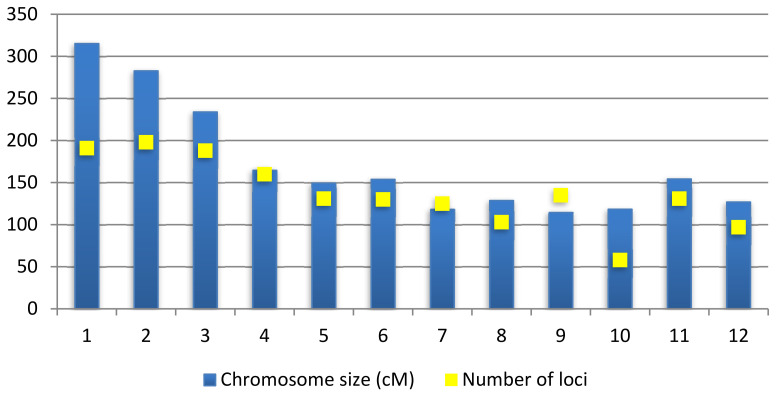
Shows the genetic size of the consensus map along with the number of loci mapped in the 12 chromosomes.

From 512 QTLs collected, chromosome 2 recorded the highest number of QTLs with 72 QTLs followed by chromosome 1 with 62 QTLs. Chromosome 12 contains only 14 QTLs, which is the least number of QTLs per chromosome. A study from Swamy et al. [46] also reported that chromosomes 1, 2 and 10 contained the highest number of QTLs for rice grain yields. There are also other studies that state that chromosome 1 has high QTL density of traits related to leaves, biomass and roots and chromosome 10 has the lowest density for the same traits [47]. This shows that the results of this consensus map are in line with previous studies where chromosome 1 and 2 contain a high number of QTLs compared to other chromosomes (Figure 2). The 512 QTLs on this consensus map spans an overall length of 2058.73 cM in this genetic map. The number of QTLs on the consensus map was reduced by 141 QTLs compared to the original 653 QTLs as BioMercator V4.2 automatically removed inverted common loci. Figure 3 shows the size of each chromosomes and the number of loci contained in these chromosomes. The number of loci can be more than the size of the chromosomes as there are some loci that overlap between each other.

This consensus map combines QTLs and molecular markers of various hybrids between several rice varieties. Several studies have used the Vandana, as it is a drought-tolerant variety. There are several QTLs related to grain yields under drought stress reported from Vandana variety such as qDTY2.3 and qDTY3.2. qDTY2.3 has a significant effect in increasing grain yield in severe stress environments, while qDTY3.2 contributes to the variation of canopy temperature during flowering and dry weight of seedlings under drought stress [48]. Variety IR58821-2-B-1-2-1 used in a study by Ali et al. [28] has root characteristics suitable for drought environments and are found to contribute many alleles that are resistant to drought. In addition, Nootripathu, which is an drought-resistant indica variety has deep and thick root systems, which is suitable for drought stressed environments [31,37]. In this study QTLs that have large effects on grain yield was inherited through allele from Nootripathu. IRAT109 is also a drought-tolerant variety found to contribute to many of the desired alleles in QTLs associated with root growth [33]. The CT9993 variety used for crossing in some studies is a variety that has deep root systems. CT9993 contributes alleles that have a significant effect on crop yields under stressful environments [34]. This variety is also said to have alleles involved in cell elongation in rice under water pressure conditions [45].

### 2.3. Meta-QTL Analysis for Frought Tolerance in Rice

The number of meta-QTL (MQTL) on each chromosome is determined based on Model selection criteria which are Akaike information content (AIC), AIC correction (AICc), AIC 3 candidate models (AIC3), Bayesian information criterion (BIC) and Average Weight of Evidence (AWE). The value considered most appropriate for conducting meta-analysis is when the model value of the selection criteria is the lowest in at least three models out of the five generated models. Molecular markers flanking the selected meta-QTLs are also identified. Table 2 shows a summary of all the meta-QTL obtained. There are 70 Meta-QTLs, with a total of 453 QTLs. There are 59 QTLs from the consensus map that were not included in the meta-QTL as the probability of such QTL membership was less than 50%. Five meta-QTL regions in chromosomes 1, 2 and 3 were selected for functional annotation as these areas contained high number of QTLs related to drought tolerance and many rice traits involved in tolerance mechanisms against abiotic stress. The first number of each MQTL names indicates the chromosome number where the MQTL is located.

For chromosome one, the AIC model showed a value of seven, while for the other four models the value was six. So a value of 6 was chosen to conduct a meta-analysis for chromosome 1 because of its majority number. Chromosome 1 contains 62 QTLs that makes up 6 meta-QTL regions. The highest QTL numbers is contained in areas MQTL 1.5 and MQTL 1.6 and have a probability of QTL membership between 67% to 100%. The properties within these meta-QTL areas are also high i.e., 6 properties on MQTL 1.5 (GY, PH, Bio, Rt, FL & SH) and 8 properties on MQTL 1.6 (GY, Bio, Lf, PH, SH, St, Rt, & FL). The lowest QTL distribution is in the MQTL 1.1, which contains only 3 QTLs and 3 rice properties (FL, Wat & Lf). MQTL 1.5 is observed in variety IR58821-2-B-1-2-1, Nootripathu, IRAT109 and CT9993 while MQTL 1.6 may be observed in Vandana, Nootripathu, IRAT109 and CT9993.

The number of meta-QTL proposed based on the criteria selection model generated for chromosome 2 is two. Chromosome 2 contains the highest number of QTLs associated with rice tolerance to drought at 53 QTLs and also includes the highest quantity of 12 rice traits out of the 13 traits studied. The probability of membership for each QTL is 100% except for some QTLs namely qGY2.2 (65%) and qFLL2.1 (63%) found on MQTL 2.1 while qRPI2.1 (56%), qPRN2.1 (56%), and qA_FS_MQM_1 (99%) were on MQTL 2.2. MQTL 2.1 and MQTL 2.2 are seen as suitable for functional annotation as the two meta-QTLs contain high QTL co-localization of 21 QTLs and 32 QTLs respectively. The area also includes many rice traits, which are 9 properties on MQTL 2.1 (St, FL, Lf, Bio, Rt, DS, Temp, Sd, & GY) and 10 properties on MQTL 2.2 (Rt, Lf, FL, RS, GY, St, PH, Temp, Bio & Wat). Variety CT9993, IRAT109 and Nootripathu contains both MQTL 2.1 and MQTL 2.2 while variety Vandana and IR58821-2-B-1-2-1 contains only MQTL 2.2.

**Figure 4:**
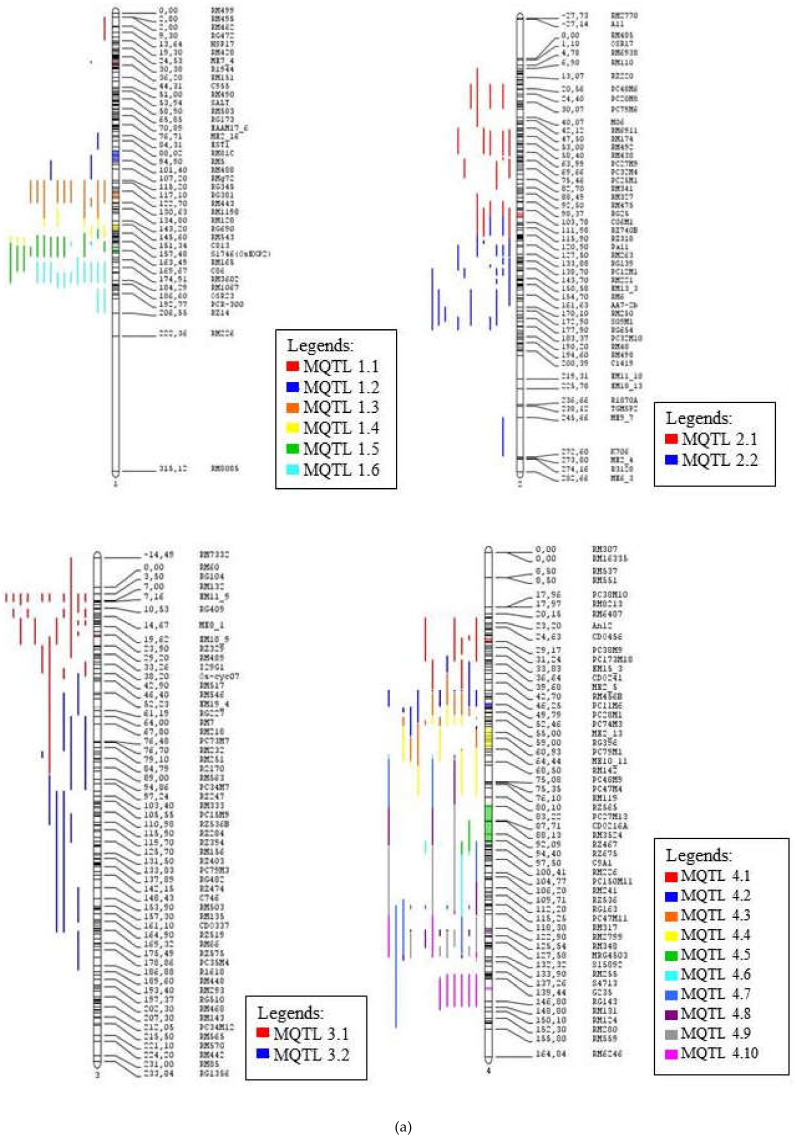

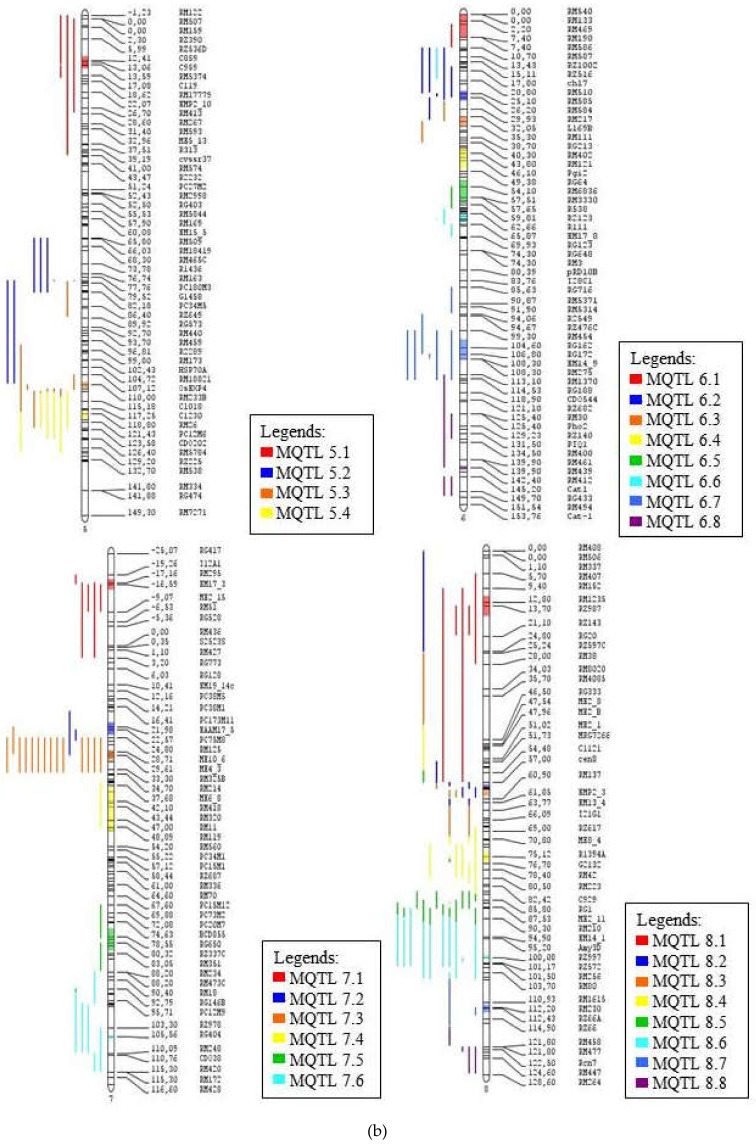

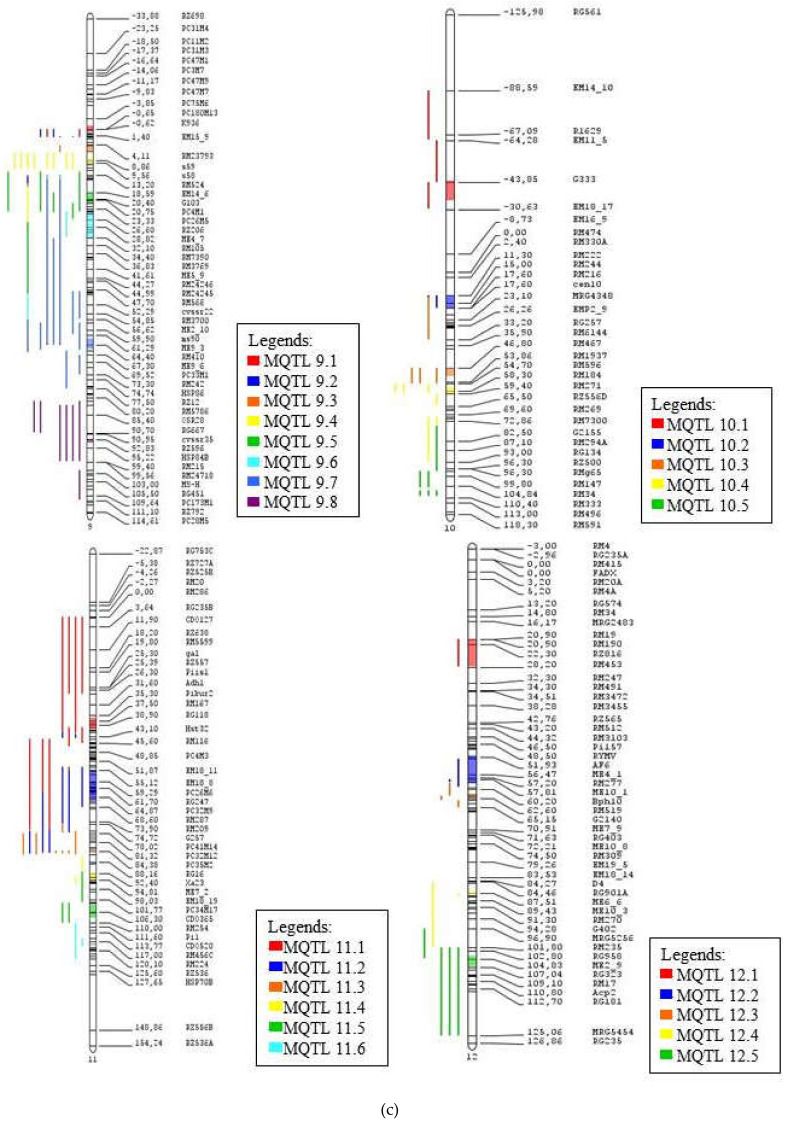
The consensus map of the QTL associated with drought tolerance following meta-analysis, (**a**) chromosome 1–4, (**b**) chromosome 5–8, (**c**) chromosome 9-12. The coloured regions represent the meta-QTL regions with reduced confidence interval. The QTL are coloured according to their respective meta-QTL regions. The clearer figures (Figures S1–S3) can be seen in the supplementary file.

**Figure 5:**
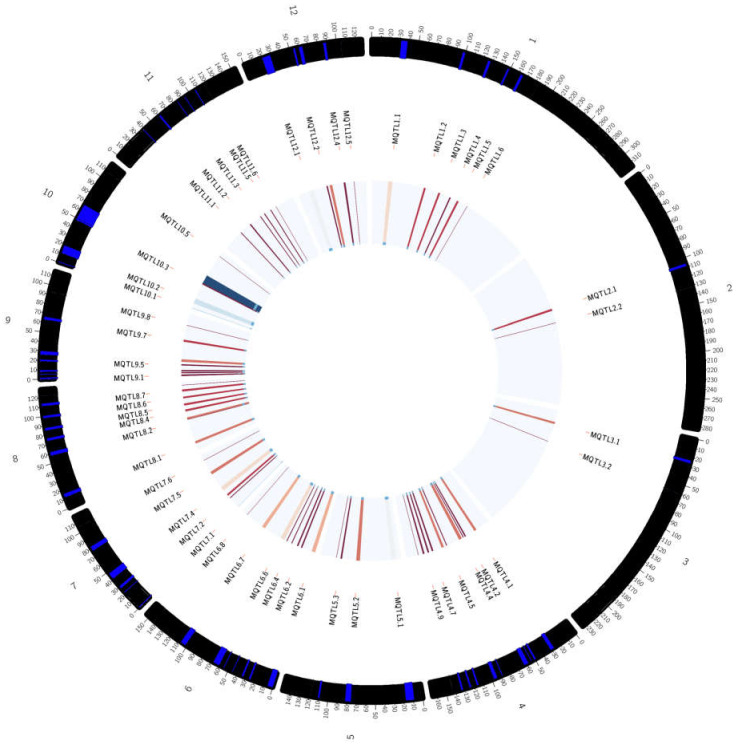
Circular representing the overall meta-QTL information including meta-QTL names, the position of the meta-QTLs in the chromosome, and the frequency of QTLs involved for each meta-QTL mapped. The outer ring indicates the position of the meta-QTLs in the chromosome, the middle circle is related to the meta-QTL names, and the centre circle reflects the frequency of QTLs involved for each meta-QTL mapped (the frequency increases from lighter to darker color—red to blue).

Chromosome 3 contains 36 QTLs that have been divided into two meta-QTL areas based on the criteria selection model. MQTL 3.1 contains 25 QTLs, which include 8 properties of rice (Rt, Lf, St, Bio, FL, Wat, GY & PH) while MQTL 3.2 contains 11 QTLs and 4 properties (Lf, Rt, FL & GY). The probability of QTL membership in MQTL 3.1 is 100% except qRPI3.1, which has a membership probability of 73%. MQTL 3.2 has a 100% membership probability for each QTL. The area of MQTL 3.1 is found to be suitable for functional annotation as this area contains a high amount of QTL, which includes a lot of rice properties. MQTL 3.1 is present in IRAT109, CT9993, Vandana and IR58821-2-B-1-2-1. Therefore, MQTL 1.5, MQTL 1.6, MQTL 2.1, MQTL 2.2 and MQTL 3.1 were chosen for functional annotation as these meta-QTL have the highest number of QTLs and rice traits compared to the other meta-QTL areas. Figure 4 shows the distribution of the MQTLs on the 12 chromosomes of rice, while Figure 5 provides an overall representation of the meta-QTL information, including the names, position on chromosome and the frequency of QTLs mapped to a particular meta-QTL.

### 2.4. Functional Annotation for Candidate Meta-QTL 

Functional annotation was conducted on MQTL1.5, MQTL1.6, MQTL 2.1, MQTL 2.2 and MQTL 3.1 The MQTL in chromosome one contains major QTL related to grain yield i.e., qDTY1.1. This particular QTL exerts positive effect under various environments with drought stress as well as stress-free environment in hybrid populations between N22 × Swarna, N22 × IR64, and N22 × MTU1010 [49]. The meta-QTL areas in chromosomes two also contain major grain yield QTLs, qDTY2.2 and qDTY2.3. The qDTY2.2 has shown significant effect on leaf rolling of MRQ74 and MR219 pyramided line population and also proves to be beneficial in improving traits related to drought tolerance [50]. MR219 has also been improved to exhibit drought-tolerance with a yield advantage of more than 1500 kg ha^−1^ and this is a result of pyramiding drought yield QTLs, qDTY 2.2, qDTY 3.1, and qDTY 12.1 through marker assisted breeding [51]. MQTL 3.1 also contains qDTY3.1 that when pyramided with others qDTYs into the rice cultivars will contribute in the increasing of grain yield over the parents plant. MR219 and MRQ74 that were pyramided with qDTY2.2, qDTY3.1, qDTY12.1 were reported to provide yield advantage of 903 to 2500 kg ha^−1^ over recipient parent MR219 and 1009 to 3473 kg ha^−1^ over recipient parent MRQ74 [50].

#### 2.4.1. MQTL 1.5

**Figure 6:**
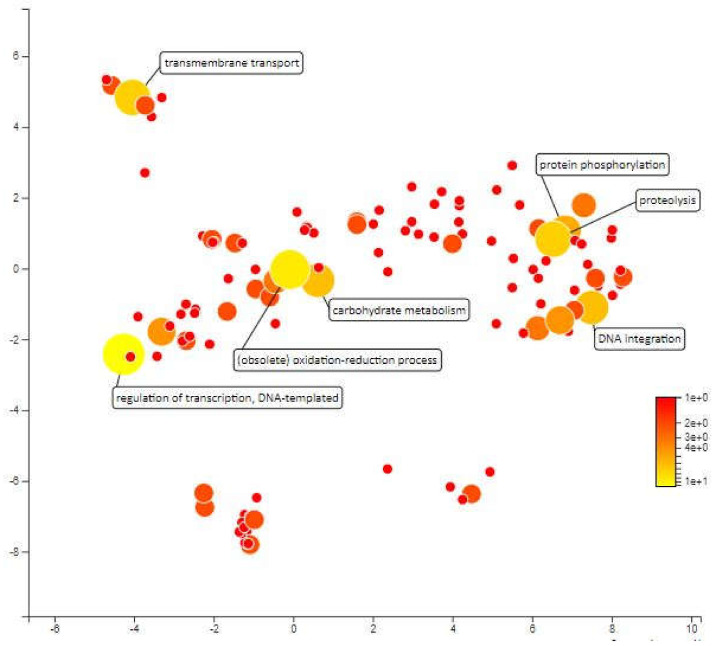
Scatterplot representing the enriched biological process gene ontology in MQTL1.5. The intensity of the color increases from red to yellow as the GO term is enriched.

MQTL1.5 has an interval of 3.17cM which covers an area of 1546kbp in chromosome 1 and contains approximately 268 annotated genes. The mapping and gene ontology annotation for these genes predicted functions such as proteolysis, transmembrane transport, redox reactions and DNA integration as the most abundant biological processes in MQTL1.5. About 18 genes were annotated for proteolytic activity. Enhanced expression of protease coding genes is normal under different abiotic stresses as there is a need for rearrangement of plant metabolism, remodeling of cell protein parts, degradation of proteins that are impaired or unwanted, and the remobilization of nutrients [52]. Low proteolytic activity and low expression of certain cysteine protease genes under water deficit pressure during the early stages of development have been considered indicators of drought-tolerant varieties for winter wheat (*Triticum aestivum* L.) cultivars [53]. There are also 18 genes that have been annotated for transmembrane transport and according to Jarzyniak & Jasiński [54], plant membrane transport systems plays an important role in adapting to water-deficient environments. The transmembrane transport genes in MQTL 1.5 areas covers ABA translocation, stomatal opening, cuticular structure, root responses and osmotic adjustment which can paves the development of new drought-tolerant varieties. Further, 13 genes were annotated for redox processes. There is much evidence to suggest the importance of redox homeostasis under saline stress and drought in which species of reactive oxygen (ROS) and antioxidants interact with the metabolic interface (metabolic interface) for signals derived from inappropriate environmental signals [55]. Furthermore, the 13 genes annotated for DNA integration and 10 genes that were annotated for DNA template regulation of transcription may not be directly related to drought tolerance. Next, there are 7 genes annotated for protein phosphorylation. This biological process has been recognized as an important mechanism for environmental stress signalling. Protein phosphorylation is protein post-translational modifications which plays a crucial role in plant stress tolerance by transmitting intracellular signals from the cell surfaces to the nucleus to regulate cellular functions of proteins (Figure 6).

There are two main genes that respond to water deficiency stress in the MQTL 1.5, namely Abscisic Acid-Insensitive Protein 5 (ABI5) and G-box binding factor 4 (GBF4). ABI5 is a basic leasing zip transcription factor that plays an important role in regulating seed germination and early seedling growth in the presence of abscisic acid and abiotic stress. The expression of ABI5 can be observed at the ends of the roots, nodes and leaf veins at the seeding stage, while in more mature plants, its activity has been shown in the edges of leaves and flowers [56]. ABI5 is also known to regulate the expression of DGAT1 (*Diacylglycerol Acyltransferase 1*) in 7-day-old seedlings if there is salt and osmotic pressure. DGAT1 encodes a major enzyme in the biosynthesis of TAG (*Triacylglycerol*), which accumulates in stress-resistant plants. The findings by Collin et al. [57] showed that HvABI5 plays a role in regulating drought response in barley. HvABI5 may be involved in the adaptation of ABA signals by the regulation of feedback between biosynthesis and signaling. In addition, HvABI5 also demonstrates different mechanisms of action in regulating the response to drought and seed germination in barley.

GBF4 is a basic leasing zip (bZIP) that binds to the G-box motif (5’-CCACGTGG-3’) and is influenced by the *rbcS-1A* gene. G-boxes motifs are cis-acting elements, which are able to influence certain plant genes exposed to various stimuli such as light induction or hormone control. GBF-type bZIP transcription factors are reported to be related to plant responses to hormones such as absicic acid (ABA), ethylene and methyl jasmonate (MeJA), which individually or collectively play an important role in plant resilience [58]. GBF4 can interact as a heterodimer with GBF3. Studies on GBF4 are still lacking but studies on GBF3 have shown that it plays a role in drought tolerance in *Arabidopsis thaliana* and the role of this gene is also perpetuated in drought tolerance and other abiotic stresses in some other plant species [59].

#### 2.4.2. MQTL 1.6

**Figure 7:**
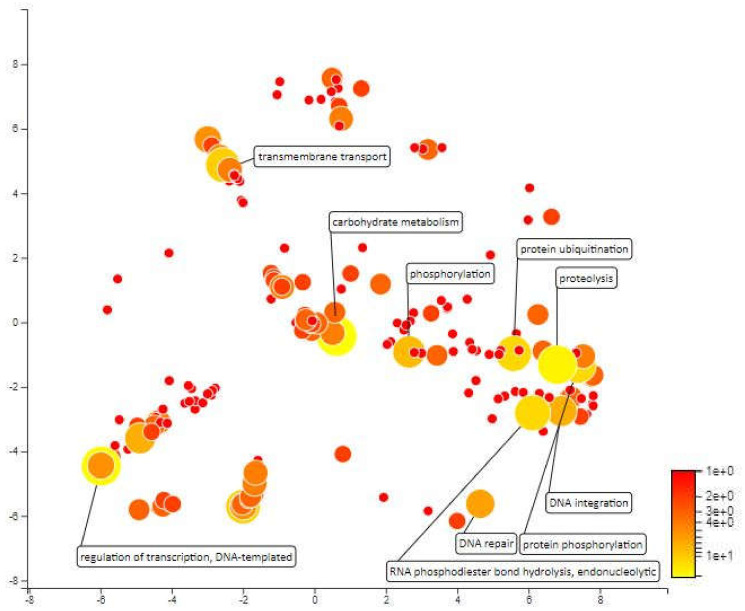
Scatterplot representing the enriched biological process gene ontology in MQTL1.6. The intensity of the color increases from red to yellow as the GO term is enriched.

The distance for MQTL 1.6 is so small that it cannot be identified by the BioMercator V4.2 software and the molecular markers that enclose this area are also a distance from each other. However, the molecular markers closest to the meta-QTL; RM431 and RM1067 were used as area markers for functional annotation. This area was selected for analysis because there are some important QTLs localized within this area, which can contribute to drought resistance. A total of 640 genes have been annotated for MQTL1.6 area. Redox processes, carbohydrate metabolic processes, proteolysis and protein ubiquitination are among the most enriched biological processes within this area. Carbohydrate metabolism in rice is influenced by drought stress, daily changes in environmental conditions and characteristics of various rice varieties [60]. Ubiquitin conjugation is a major regulator of stress-responsive transcription factors and other regulatory proteins. By modulating the amount and activity of regulatory proteins, ubiquitination plays an important role in regulating transcriptional changes required for adaptation to abiotic stress [61] (Figure 7).

Based on the mapping and annotations carried out, there are three main genes that respond to water deficiency in MQTL1.6. They are the protein kinase PINOID (PID), histidine kinase 2 (AHK2) and the autophagy related protein 18A (ATG18A). PID genes can be induced by auxins and clones of this gene is found to encode protein-serine/threonine kinase [62]. This protein kinase is found in primordia cotyledons, leaves and flowering organs as well as in vascular tissue in developing organs or adjacent to meristems. High PINOID expression under the control of constitutive CaMV 35S promoter causes phenotypes observed in mutants that have altered sensitivity to auxin transport. Transport and regulation of auxin is important for the growth and development of roots in plants. Since roots are among the plant organs that play an important role in the response to dehydration stress, these PINOID genes are among the important genes that respond to drought stress.

Histidine kinases (HK) are transferase class enzymes that play a role in signal transduction between cells. HK in plants operate through His-Asp phosphorus and control many physiological processes and developments throughout the plant’s life cycle. In Arabidopsis, HK is involved in the signaling functions of ethylene, osmosensing, cytokines, mega-gametophyte expansion, cold perception, regulating salinity sensitivity, response in drought stress and resistance to bacterial and fungal infections [63]. The study also states that the loss of *ahk2* function, single mutant *ahk3* and double mutant *ahk2-ahk3* showed high tolerance to drought and salinity. This indicates that AHK2 and AHK3 serve as negative regulators of salt and osmotic pressure. Expression analysis showed little or no change in the expression of *AHK2* and *CRE1* genes in various abiotic stresses in both root and shoot tissues. AHK2 and AHK3 were found to negatively control the osmotic pressure response in Arabidopsis. *CRE1* also regulates osmotic pressure negatively in the presence of cytokines [64].

Autophagy is a process of protein degradation in which cells will recycle the contents of the cytoplasm when undergoing environmental stress or at a certain stage of development. In plants, autophagy is caused by abiotic stress including nutrient deficiency and oxidative stress. A study by Liu et al. [65] showed induction of autophagy in high salinity and osmotic pressure is equivalent to increased gene expression associated with autophagy (*AtATG18a*). As a result, autophagy-defective RNAi-AtATG18a plants are more sensitive to salinity and drought stress than wild-type crops. This indicates that autophagy responds to abiotic stress. MdATG18a an ATG18a homologs from *Malus domestica* are highly expressed in apple crops, increasing its tolerance to drought stress [66]. This is caused by greater autophagosome production and higher autophagic frequency. These processes can help lower protein aggregation and limit oxidative damage. Thus, overexpression of MdATG18a may increase drought tolerance by increasing autophagy, which is involved in decreasing oxidized proteins and regulating ROS levels under drought stress.

#### 2.4.3. MQTL 2.1

**Figure 8:**
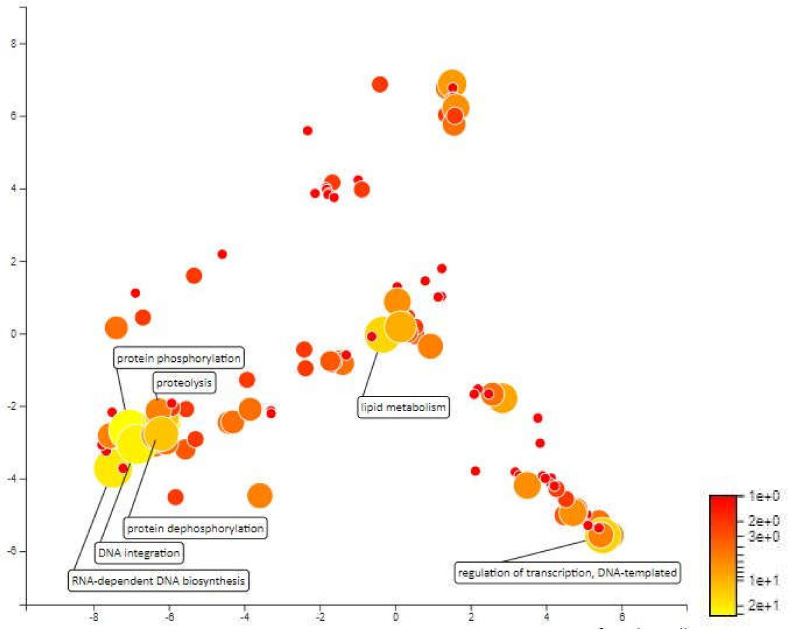
Scatterplot representing the enriched biological process gene ontology in MQTL2.1. The intensity of the color increases from red to yellow as the GO term is enriched.

MQTL 2.1 is located within an interval of 3.39 cM on chromosome 2. The physical distance of the MQTL is 2135kbp. 319 genes were annotated in this meta-QTL. Protein phosphorylation, DNA integration and RNA-dependent DNA biosynthetic process are three most prominent biological processes based on Blast2GO annotation. This region is relatively small and based on MQTL annotation this region did not show presence of any genes involved in the regulation of drought stress in rice. However, there is one locus linked to abscisic acid (ABA), which is known to regulates plant’s responses to dehydration and optimized water use. The mitochondrial transcription termination factor (MTERF) in located in this locus. This protein is involved in regulating the expression of mitochondrial genes and are closely related to the function of the mitochondrion and chloroplast [67]. The mTERF protein responses towards abiotic stress through disruption of ABA retrograde signaling that results from a disruption in chloroplast homeostasis. The study also states that gene from this protein may improve tolerance of *Arabidopsis thaliana* to salt and osmotic stress and also alter sugar responses during seedling establishment, likely as a consequence of decreased sensitivity to ABA [68]. Furthermore, the promoter sequences of mTERFs in *Capsicum annuum* were found to contain abiotic stress-related elements, hormone regulation-related cis-elements, such as those responsive to auxin, MeJA(Methyl Jasmonate), gibberellin, abscisic acid, anoxic conditions, and salicylic acid, as well as osmotic stress-related cis-elements [67]. This further shows that mTERF protein may increase plant tolerance towards drought stress (Figure 8).

#### 2.4.4. MQTL 2.2

**Figure 9:**
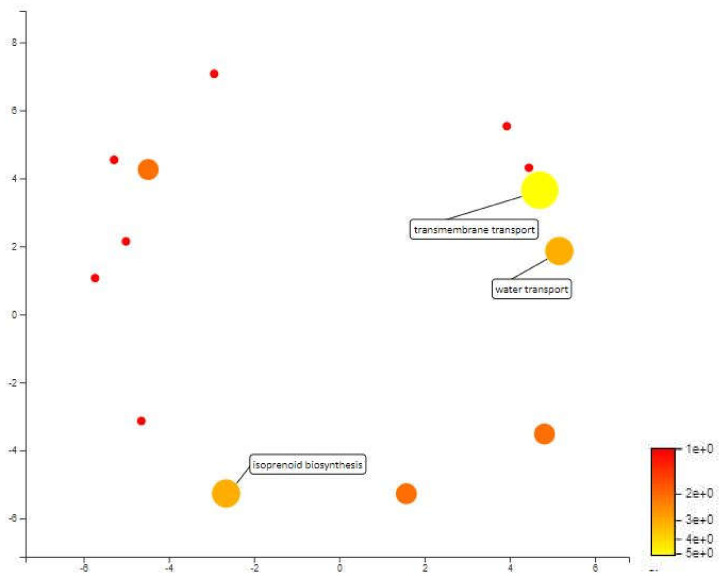
Scatterplot representing the enriched biological process gene ontology in MQTL 2.2. The intensity of the color increases from red to yellow as the GO term is enriched.

MQTL 2.2 is a 112kbp area in chromosome 2 and only contains 19 annotated genes. Based on the mapping and annotation, the most important biological process in this area is transmembrane transport with 5 annotated genes involved in water transport and isoprenoid biosynthetic process. The water transport annotated genes in MQTL 2.2 are aquaporin and aquaporin PIP 1-2 which are also significant genes related to drought response. Aquaporins are said to play an important role in plants defense responses against biotic and abiotic stressors. It is also said that water uptake and transcellular water flow in roots are largely mediated by aquaporins PIP [69]. According to Alexandersson et. al and Jang et. al, subfamilies of aquaporins PIPs are most responsive to drought stress and most of them undergo transcriptional down-regulation and only some are up-regulated [70,71]. In rice, the expression of OsPIP1-1 and OsPIP1-2 was up-regulated when osmotic stress was artificially induced by 10% polyethylene glycol (PEG) [72]. The study also stated that OsPIP1-2 play an essential role in water transport in rice leaves. Furthermore, OsPIP1-2 along with OsPIP1-1, OsPIP2-1, OsPIP2-4 and OsPIP2-5 were expressed in roots and leaves. Thus balancing water homeostasis in roots and leaves of rice plants may need the coordination of these OsPIP proteins (Figure 9).

Isoprenoid biosynthetic is annotated for heterodimeric geranylgeranyl pyrophosphate synthase small subunit protein. The protein works with large subunit (GGPPS1) which catalyzes mainly the production of geranygeranyl-diphosphate in vitro [73]. The protein is also associated with monoterpene biosynthesis. Monoterpenes which is a isoprenoid is a volatile organic compound which has shown increase in plants during water deficit condition [74,75]. Monoterpenes have been shown to protect leaf membranes from oxidation and to increase heat stress resistance by modification of the leaf thermal tolerance. It is also an indicator for expression of genes that respond to water stress condition [76].

#### 2.4.5. MQTL 3.1

MQTL 3.1 is the biggest selected MQTL area which has an interval of 3.58 cM that forms an area of 486 kbp in chromosome 3. The area contains 787 annotated genes. Redox processes, regulation of transcription, protein phosphorylation, transmembrane transport and carbohydrate metabolic processes are the most dominant biological processes annotated by the Blast2GO in MQTL 3,1. In this meta-QTL area, there are two genes that are annotated for biological process that respond to osmotic stress which are Protein Detoxification 48 and inositol-tetrakisphosphate 1-kinase 2.

**Figure 10:**
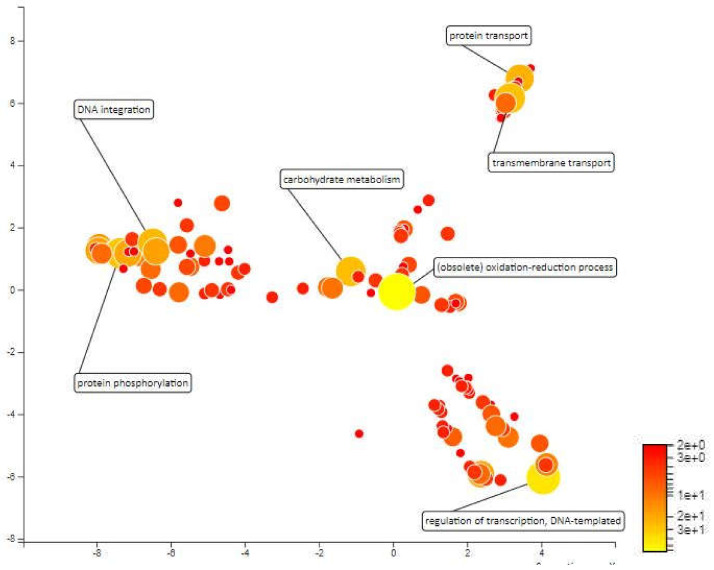
Scatterplot representing the enriched biological process gene ontology in MQTL3.1. The intensity of the color increases from red to yellow as the GO term is enriched.

Detoxification (*DTX48*) is one of the detoxification efflux carriers/multidrug and toxic compound extrusion (*DTX/MATE*) gene. This gene is significant in the translocation of abscisic acid (ABA), a phytohormone with profound role in plants under various abiotic stress conditions. Overexpression of *Gh_D06G0281* (*DTX/MATE*) gene from cotton in Arabidopsis line shows high tolerance towards drought, salt, and cold stress with high production of antioxidant enzymes and significantly reduces levels of oxidants. Furthermore, as a measure of cell membrane stability compared to wild types, transgenic plants that contained the *DTX/MATE* genes displayed altogether higher relative leaf water content, decreased excised leaf water loss and a substantial decrease in ion leakage [77]. In addition, *dtx50* mutant plants were more tolerant to drought with lower stomatal conductance, consistent with its function as an ABA efflux carrier in guard cells compared to wild type plant according to Zhang et al. study [78] (Figure 10).

Inositol-tetrakisphosphate 1-kinase 2 (ITPK2) is a kinase that can phosphorylate various inositol polyphosphate such as Ins(3,4,5,6)P4 or Ins(1,3,4)P3 and participates in phytic acid biosynthesis in developing seeds. Phytic acid is the primary storage form of phosphorus in cereal grains and other plant seeds [79]. The study also shows that a high expression of GmITPK2 isoform in soybean as an early dehydration responsive gene was induced by dehydration and exogenous treatment with ABA. Moreover, a few genes that are related to osmotic adjustment and reactive oxygen species scavenging were down-regulated in the mutant and overexpression lines of *OsITPK2* gene. A study by Du et. al. (2011) also stated that *OsITPK2* is an important member of the OsITPK family for stress responses, and an optimal expression level is essential for drought and salt tolerance in rice [80].

These proteins identified are basic leucine zipper transcription factor (bZIP) and kinase proteins which are regulatory proteins involved in regulating signal transduction and gene expression against drought stress. bZIP provides resistance to plants facing severe environmental stress and plays an important role in every phase of the plant growth process [81]. Protein kinase works to regulate the response to stress by adjusting plant activity or response through phosphorylation of target proteins. Drought stress will activates signal to synthesize protein kinases and transcription factors, will activate responses and other signals such as changes in the expression of genes, proteins and enzymes. These signals in turn will result in changes in metabolic processes in plants such as antioxidant activation and synthesis, osmoprotectant synthesis and accumulation as well as stomata closure which further helps plants cope with drought [82] (Figure 10).

## 3. Materials and Methods

### 3.1. Bibliographic Review and Data Synthesis

Information about QTLs that are related to drought tolerance in rice and molecular markers from the genetic maps was collated from published reports. There were 20 reports of QTL mapping for rice traits under drought including this study. The QTL are from different traits from different studies and few of them being in similar positions. Total of 635 QTLs were collected from previous studies that include various traits of rice reported in drought studies. These traits involved roots, leaf, flower, grain yield, plant height, shoot, water, drought score, biomass, temperature, recovery score, stem and seed function that may be affected by the change in water potential within the plant. Only QTLs for traits under drought stress condition from the studies were included in the data synthesis while the QTLs for traits observed under control conditions were excluded, as these QTLs are not relevant to the present investigation. All the information of QTLs and molecular markers from each past study was mapped and visualized using Biomercator V4.2.

### 3.2. Consensus Map and QTL Projection

Total of 27 genetic maps from 20 previous studies were used to construct consensus genetic map using Biomercator V4.2. The rice genetic linkage map of Temnykh et al. (2001) was used as a reference map [83]. The markers and QTL from 20 studies were integrated into the reference map to develop a consensus map. Inversions of marker sequences were filtered out. The projection of QTLs on the genetic map was based on LOD scores, phenotypic variation explained by each QTL, confidence intervals and QTL positions. Confidence interval of 95% (CI) was calculated based on equation from Darvasi and Soller (1997) which is $=\;\frac{530}{NR^{2}}$, where N is the population size and R2 is the proportion of phenotypic variance of QTL [84].

### 3.3. Meta-QTL Analysis

Following generation of consensus map and QTL projection, analysis was conducted to get the meta-QTLs. Meta-QTLs are QTLs that are integrated from QTLs that are collected from past studies. The number of meta-QTLs (MQTLs) on each chromosome is determined from different experiments based on AIC (Akaike information content), AICc (AIC correction), AIC3 (AIC 3 candidate models), BIC (Bayesian information criterion) and AWE (average weight of evidence). The number that was chosen to carry out meta-analysis is when the values of the model selection criteria were the lowest in at least three out of five models [85]. The positions and 95% confidence intervals of each MQTL were calculated. The meta-analysis was also performed using Biomercator V4.2.

### 3.4. Functional Annotaion

Once meta-QTL analysis is performed, functional annotation was conducted on the selected Meta-QTLs for further study. Molecular markers that flank the selected Meta-QTLs were identified and the physical position of the molecular markers was obtained through Gramene Marker Database. Next, the position range between the molecular markers that flank the Meta-QTL is inputed into the search field in the Rice Genome Browser under the MSU-Rice Genome Annotation Project Database and Resource (http://rice.plantbiology.msu.edu/, accessed on 1 April 2021) for locus ID information, FASTA sequence and annotated data. The FASTA sequence of locus id within the area of Meta-QTL was then uploaded to BLAST2GO software (https://www.blast2go.com/, accessed on 1 April 2021). BLAST is performed to obtain gene descriptions and annotations by searching the same sequence of sequences in the query. NCBI Blast is a database selected in Blast2GO for the BLAST process Mapping process of the genes were carried following BLAST process for the entire Meta-QTL region. Mapping is the process of obtaining gene ontology terms related to descriptions obtained through BLAST. After mapping process completed, annotation of the gene ontology is carried out. GO annotation is the process of selecting gene ontology terms from a group of gene obtained through the mapping process conducted towards query sequence. This method is the deepest functional annotation in Blast2GO. This allows the most specific annotations to be determined with a certain level of reliability. Parameters are set to ‘default’ before annotation.

## 4. Conclusions

A total of 653 QTLs for drought tolerance in rice from 27 genetic maps were recorded for analysis. The QTLs collected include QTLs related to the 13 traits of rice that respond to drought i.e., grain yield (GY), drought score (DS), plant height (PH), biomass (Bio), roots (Rt), shoots (Sh ), leaves (Lf), water (Wat), temperature (Temp), flowers (FL), recovery score (RS), stem (St) and seeds (Sd). Through the use of BioMercartor V4.2 software, a consensus map was generated from 27 genetic maps that had been extracted and there are 512 QTLs on this consensus map with the overall length of this genetic map being 2058.73 cM. 

Then, meta-QTL analysis was performed using the same software. The total meta-QTL generated is 70 MQTL, which contains a total of 453 QTL. Five meta-QTLs from Chromosome 1, 2 and 3 were selected as these areas contain a high number of QTLs and include many traits in rice that responds to drought. Out of five MQTL, four areas, which are MQTL 1.5, MQTL 1.6, MQTL 2.2 and MQTL 3.1 are related to major qDTY which are qDTY1.1, qDTY2.2, qDTY2.3 and qDTY3.1. MQTL 1.5 that flanked by RG109—RM315 in chromosome one and has a small genetic distance of 3.17cM. Thus, this area may be suitable for use in Marker Assisted Selection (MAS) and pyramiding of QTLs for yield and drought tolerance in rice. 

## 5. Patents

The consensus genetic map and the MQTL maps are protected under LY 2020005591.

## Figures and Tables

**Table 1:** Information derived from previous studies on drought in rice.

Title	Parents	Type of Crossing	Number of Marker Used	Type of Marker	Population Size	Number of QTLs	References
Mapping QTLs for root traits in a recombinant inbred population from two indica ecotypes in rice	IR58821–23-B-1–2-1 X IR52561-UBN-1–1-2	RIL	367	AFLP, RFLP	166	39	[28]
A large-effect QTL for grain yield under reproductive-stage drought stress in upland rice	Vandana X Way Rarem	SF3	126	SSR	436	22	[29]
Locating QTLs controlling constitutive root traits in the rice population IAC 165 × Co39.	IAC 165 X CO39	RIL	182	RFLP, SSR	125	42	[30]
Molecular mapping and location of QTLs for drought-resistance traits in indica rice (*Oryza sativa L.)* lines adapted to target environments	Nootripathu X IR20	RIL	79	SSR	250	21	[31]
Using chromosome introgression lines to map Quantitative Trait Loci for photosynthesis parameters in rice (*Oryza sativa* L.) leaves under drought and well-watered field conditions.	Haogelao X Shennong265	BC	130	SSR	94	29	[32]
Identification of QTLs controlling rice drought tolerance at seedling stage in hydroponic culture.	IRAT109 X Akihikari	BC	57	SSR	106	5	[33]
Genetic analysis of rainfed lowland rice drought tolerance under naturally-occurring stress in eastern India: Heritability and QTL effects.	CT9993 X IR62266	DH	315	AFLP, RFLP, SSR	154	18	[34]
Quantitative Trait Loci Associated with Drought Tolerance at Reproductive Stage in Rice.	CT9993 X IR62266	DH	332	AFLP, RFLP, SSR	154	69	[19]
Saturation mapping of QTL regions and identification of putative candidate genes for drought tolerance in rice.	CT9993 X IR62266	DH	400	AFLP, RFLP, SSR	154	42	[35]
Grains yield QTLs with consistent-effect under reproductive- stage drought stress in rice.	KaliAus X IR64 KaliAus X MTU1010	BC	4	SSR	300	3	[36]
Mapping consistent rice (*Oryza sativa* L.) yield QTLs under drought stress in target rainfed environments.	Nootripathu X IR20	RIL	79	SSR	397	2	[37]
Identification and mapping of QTLs associated with drought tolerance traits in rice by a cross between Super Basmati and IR55419-04.	IR55419-04 X Super Basmati	SF2	73	SSR	418	21	[38]
Genetic variation, linkage mapping of QTL and correlation studies for yield, root, and agronomic traits for aerobic adaptation.	MASARB25 X Pusa Basmati 1460 MAS26 X HKR47	SF2	50	SSR	250	35	[39]
Genetic mapping of quantitative trait loci for grain yield under drought in rice under controlled greenhouse conditions.	Vandana X Cocodrie	SF2	88	InDels. SNP, SSR	187	6	[18]
Identification of Quantitative Trait Loci for ABA sensitivity at seed germination and seedling stages in rice.	IRAT109 X Zhenshan 97	RIL	220	SSR	154	17	[40]
Identification of quantitative trait loci for four morphologic traits under water stress in rice (*Oryza sativa* L.).	IRAT109 X Zhenshan 97	RIL	220	SSR	180	36	[41]
Genetic Basis of Drought Resistance at Reproductive Stage in Rice: Separation of Drought Tolerance From Drought Avoidance.	IRAT109 X Zhenshan 97	RIL	220	SSR	180	12	[42]
Genetic analysis for drought resistance of rice at reproductive stage in field with different types of soil.	IRAT109 X Zhenshan 97	RIL	220	SSR	180	53	[43]
Locating genomic regions associated with components of drought resistance in rice: comparative mapping within and across species.	CT9993 X IR62266	DH	315	AFLP, RFLP, SSR	154	41	[44]
Comparison of QTLs for rice seedling morphology under different water supply conditions.	Azucena X IR64	DH	189	RFLP, SSR	96	12	[45]

**Table 2:** Table shows a summary of 70 meta-QTL obtained from the analysis of all chromosomes. The highlight MQTL indicates the MQTL that has been selected for the functional annotation.

Meta-QTL	Position (cM)	Flanking Markers	Distance (cM)	Number of QTL	Number of Traits	Traits Involved
MQTL 1.1	34.57	RM522–RG394	6.84	3	3	FL, Wat, Lf
MQTL 1.2	98.65	RM5–RM34B	3.2	5	4	Lf, Bio, Temp, Rt
MQTL 1.3	126.31	RM1216–RM1198	2.82	12	6	Rt, Lf, GY, DS, FL, Sd
MQTL 1.4	147.97	RM543–RM3825	2.4	8	4	Rt, FL, Bio, PH
MQTL 1.5	163.7	RG109–RM315	3.17	17	6	GY, PH, Bio, SH, Rt, FL
MQTL 1.6	182.77	RM431–RM1067	*	17	8	GY, Bio, Lf, PH, SH, St, Rt, FL
MQTL 2.1	107.52	G45–RM6379	3.39	21	9	St, FL, Lf, Bio, Rt, DS, Temp, Sd, GY
MQTL 2.2	132.4	RM5470–RG139	*	32	10	Rt, Lf, FL, RS, GY, St, PH, Temp, Bio, Wat
MQTL 3.1	24.41	RZ329–C63	3.58	25	8	Rt, Lf, St, Bio, FL, Wat, GY, PH
MQTL 3.2	59.9	RG396–RG227	*	11	4	Lf, Rt, FL, GY
MQTL 4.1	29.02	PC33M4–RM5953	4.47	8	3	Bio, Rt, Lf
MQTL 4.2	49.97	PC11M12–RM185	1.14	6	2	Bio, Rt
MQTL 4.3	54.59	PC38M7–ME2_13	1.14	6	2	Rt, FL
MQTL 4.4	60.77	PC36M3–ME10_11	4.76	4	3	GY, Rt, Lf
MQTL 4.5	88.6	PC27M13–EMP3_10	1.56	1	1	PH
MQTL 4.6	95.9	ME9_4–C335	4.44	3	2	FL, Rt
MQTL 4.7	116.17	RM470–RM303	1.52	6	2	Lf, Rt
MQTL 4.8	124.22	RG939–RM348	1.65	3	2	FL, Rt
MQTL 4.9	132.98	R2017–R0874	1.87	7	3	FL, Rt, Bio
MQTL 4.10	142.86	G235–RG143	*	6	3	Bio, Rt, Lf
MQTL 5.1	13.05	RZ536D–C119	9.03	4	4	GY, FL, Rt, Bio
MQTL 5.2	79.08	RM430–R2217	5.43	8	5	PH, Bio, Lf, Temp, Rt
MQTL 5.3	110.1	RM233B–RM421	1.74	6	5	FL, St, Rt, Bio, Wat
MQTL 5.4	119.36	RM26–RG119	*	6	4	Sd, Lf, FL, Bio
MQTL 6.1	3.4	RM540–RM190	5.87	1	1	Bio
MQTL 6.2	25.86	Amp5–RM217	1.78	7	2	Rt, Bio
MQTL 6.3	33.84	L169B–RM111	2	6	4	GY, FL, Lf, Bio
MQTL 6.4	45.36	RM557–RG64	1.42	1	1	GY
MQTL 6.5	55.71	RM136–CD096	1.37	4	4	FL, Lf, GY, Bio
MQTL 6.6	64.21	R111–EM17_8	6.94	4	2	PH, Bio
MQTL 6.7	105.22	RM454–RM275	5.95	9	5	Lf, Rt, Bio, FL, GY
MQTL 6.8	142.5	RM439–RM141	*	5	5	Bio, Wat, temp, SH, DS
MQTL 7.1	−16.55	T12A1–ME2_15	9.7	5	4	Lf, Rt, Bio, PH
MQTL 7.2	22.33	RZ448–RM125	1.66	2	2	Rt, Bio
MQTL 7.3	29.67	ME10_6–RM8010	2.67	16	6	FL, Lf, PH, GY, Rt, Bio
MQTL 7.4	44.34	ME6_8–RM2878	7.23	1	1	Rt
MQTL 7.5	79.42	PC34M16–RM351	5.31	2	2	Bio, Rt
MQTL 7.6	105.9	RG404–RM248	0.66	6	5	Bio, FL, 2 Rt, Lf, St
MQTL 8.1	13.8	RM152–RZ143	4.43	8	4	Lf, FL, St, Bio
MQTL 8.2	57.89	RM126–ME5_3	0.59	5	4	Lf, Rt, St, FL
MQTL 8.3	59.77	MRG2181–RM137	3.92	4	3	FL, GY, Rt
MQTL 8.4	75.37	RM6032–G2132	2.74	6	6	Temp, Wat, DS, GY, Rt, FL
MQTL 8.5	86.89	G187–ME2_11	2.81	7	3	FL, GY, Bio
MQTL 8.6	99.96	RZ997–RZ572	2.93	11	3	PH, FL, Rt
MQTL 8.7	112.54	RM1615–RZ66	3.16	2	1	FL
MQTL 8.8	122.49	RM477–Rcn7	*	6	5	FL, Lf, St, Bio, Sd
MQTL 9.1	−0.61	PC75M6–PC75M4	0.91	2	1	FL
MQTL 9.2	1.71	ME2_17–C711	1.47	6	2	FL, Rt
MQTL 9.3	5.35	PC32M13–S55	1.17	1	1	Bio
MQTL 9.4	9.03	S55–PC12M5	2.39	8	5	FL, GY, Lf, Bio, PH
MQTL 9.5	20.2	EM14_6–RG553	1.41	5	4	Rt, Lf, Bio, Wat
MQTL 9.6	28.23	RM296–RM105	3.66	1	1	Lf
MQTL 9.7	64.11	ME9_3–RM257	2.87	18	5	PH, FL, Bio, Rt, Lf
MQTL 9.8	93.31	RZ596–HSP84B	*	8	5	Bio, GY, DS, Rt, FL
MQTL 10.1	−39.67	G333–EM18_17	10.36	3	3	Bio, GY, FL
MQTL 10.2	14.52	RM222–RM216	9.56	1	1	FL
MQTL 10.3	49.1	RM467–RM1937	2.21	5	4	PH, FL, Bio, Lf
MQTL 10.4	57.73	RM596–RM271	16.02	5	4	Bio, DS, Rt, St
MQTL 10.5	107.67	RM34–RM333	*	6	4	FL, GY, Lf, PH
MQTL 11.1	38.41	RM6894–RM552	1.15	11	4	Bio, Rt, Lf, FL
MQTL 11.2	60.79	PC4M2–PC33M2	1.62	4	3	Bio, Rt, St
MQTL 11.3	84.53	PC35M2–RM21	0.87	6	3	Lf, Bio, Rt
MQTL 11.4	93.64	Xa23–ME7_2	1.14	1	1	Rt
MQTL 11.5	106.12	RM206–RM254	1.14	3	3	PH, Temp, FL
MQTL 11.6	118.17	RM5926–C950	*	2	2	FL, Rt
MQTL 12.1	24.55	RM19–RM453	9.2	1	1	FL
MQTL 12.2	55.15	AF6 –RM28130	1.61	1	1	GY
MQTL 12.3	62.19	RM260–RM519	4.55	4	2	Bio, Rt
MQTL 12.4	87.5	CD0344–RG901	2.48	3	2	Rt, Bio
MQTL 12.5	105.4	RG958–RG323	*	5	5	Bio, PH, Lf, FL, Rt

GY (Grain Yield), DS (Drought Score), PH (Plant Height), Bio (Biomass), Rt (Roots), Sh (Shoots), Lf (Leaf), Wat (Water), Temp (Temperature), FL (Flowers), RS (Recovery Score), St (Stems), Sd (Seeds). * Indicates that the distance of the MQTL is too small to be identified by the BioMercator V4.2 software.

## Supplementary Materials

The following are available online at https://www.mdpi.com/article/10.3390/plants10040716/s1, Figure S1: The consensus map of the QTL associated with drought tolerance following meta-analysis, Meta-QTL, QTL and markers for chromosome 1–4, Figure S2: Meta-QTL, QTL and markers for chromosome 5–8, Figure S3: Meta-QTL, QTL and markers for chromosome 9–12.
