# Supplementary material for: Meta-Analysis of Quantitative Traits Loci (QTL) Identified in Drought Response in Rice (Oryza sativa L.)
Source: Plants (Basel). 2021 Apr 7;10(4):716. doi: 10.3390/plants10040716 (PMC8067883; doi:10.3390/plants10040716)
Supplement: Supplementary file 1 [file plants-10-00716-s001.pdf]

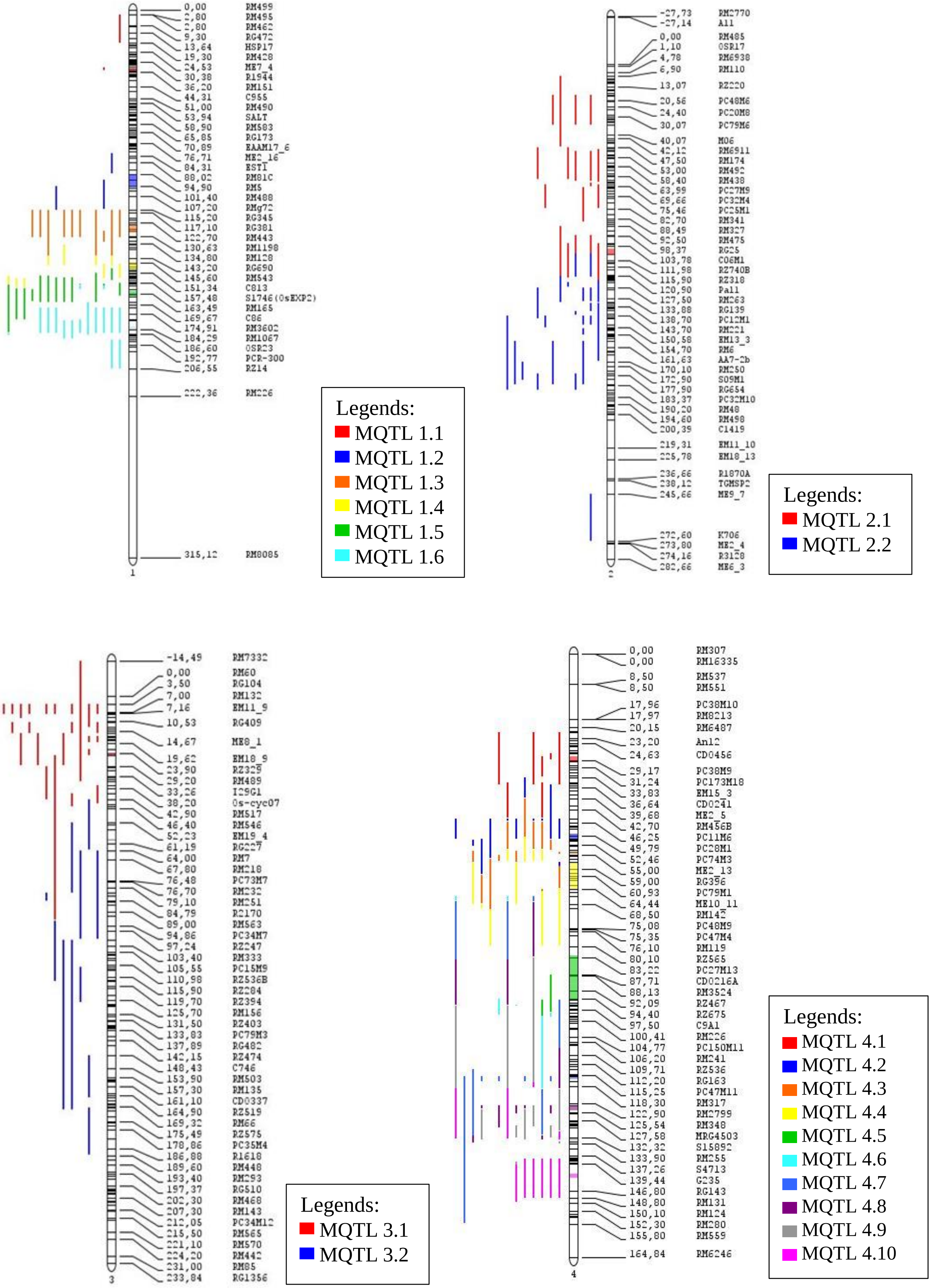

Figure 1 : Meta-QTL, QTL and markers for chromosome 1-4

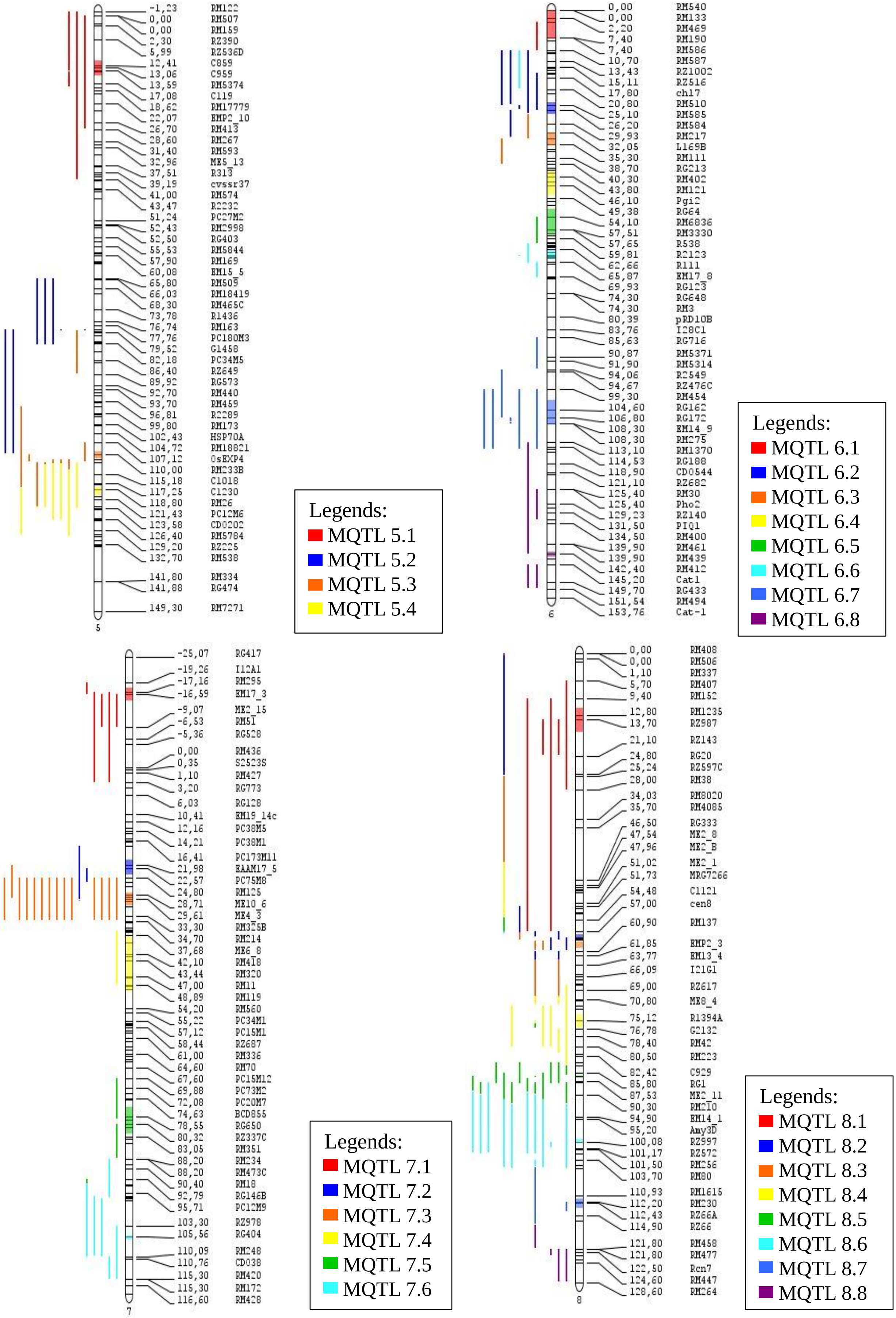

Figure 2 : Meta-QTL, QTL and markers for chromosome 5-8

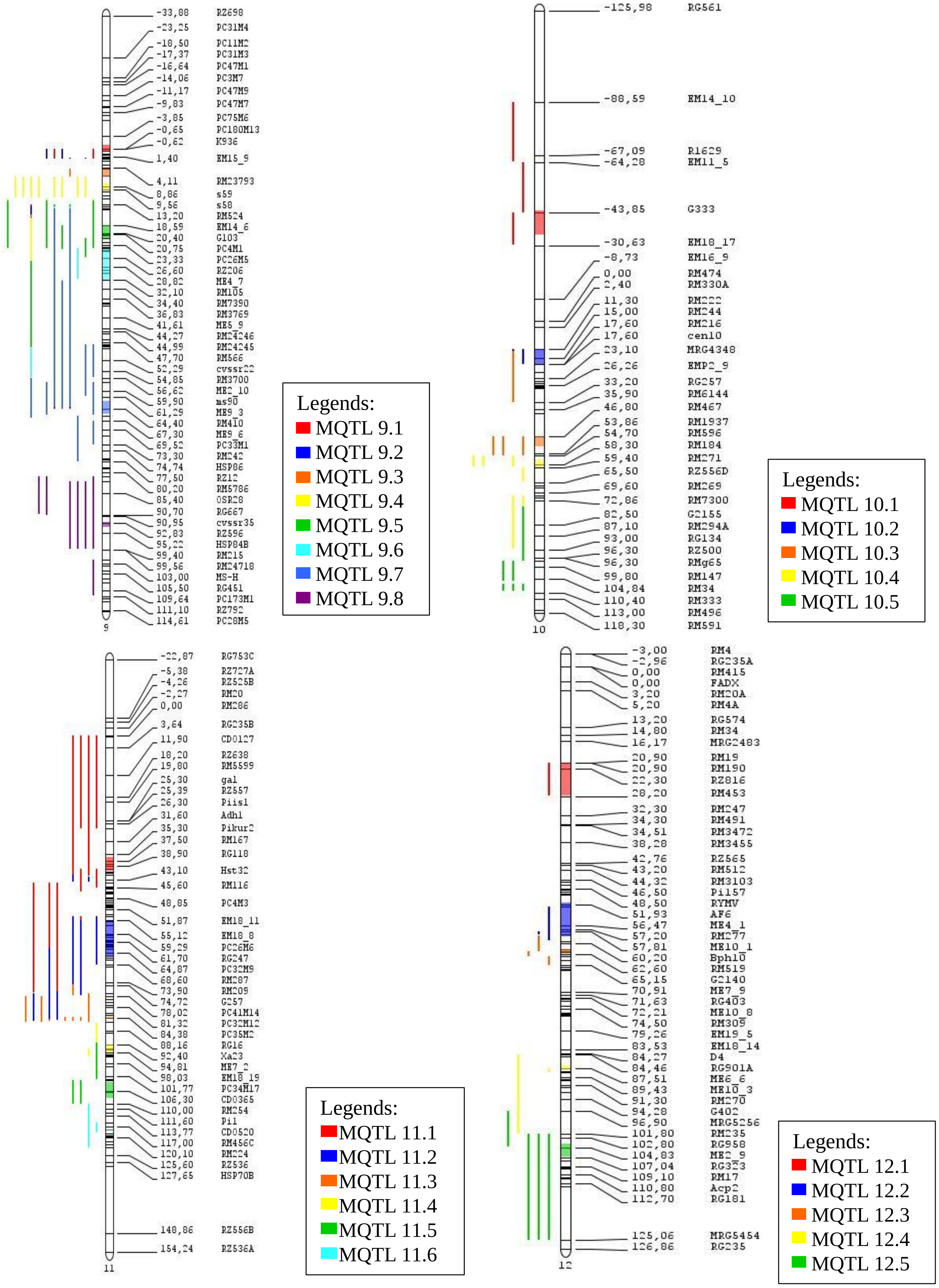

Figure 3 : Meta-QTL, QTL and markers for chromosome 9-12

Details about the map

- The colored line on the left representing the QTL corresponds to the color of shaded region on the chromosome that represent the meta QTL region of drought tolerent in *Oryza sativa.*
- The IDs on the left represent the molecular markers.
- The numbers beside the molecular markers represent the positions of the molecular markers.

Candidate meta QTL region

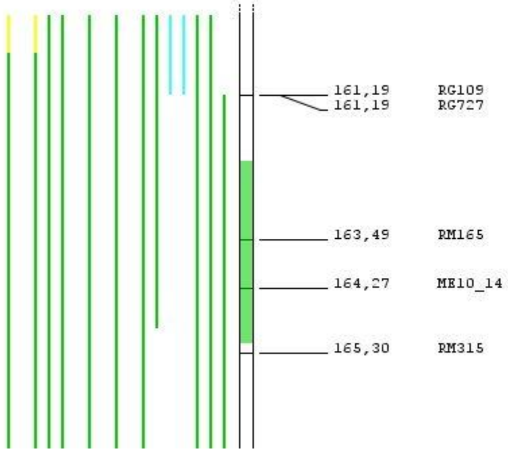

The green region represents MQTL1.5 which has the maximum number of QTL associated with drought tolerance in rice. The genetic distance of this MQTL is a 3.17 cM and flanked by RG109 and RM 315

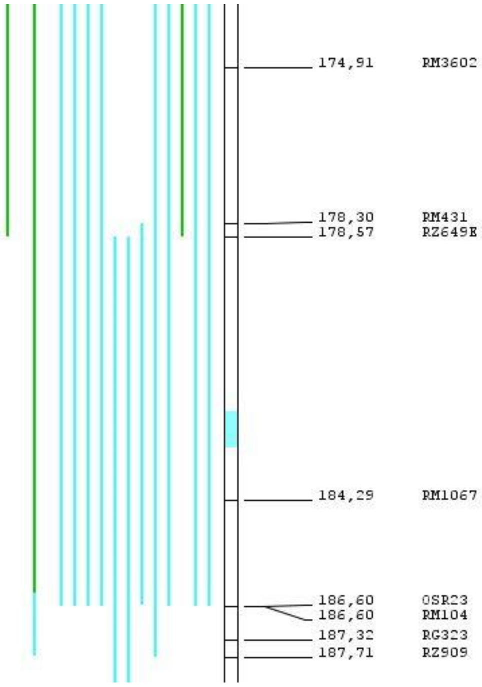

The light blue region represents MQTL1.6 which has the maximum number of QTL associated with drought tolerance in rice. The genetic distance of this MQTL is a 1.4 cM and flanked by RM 431 and RM 104

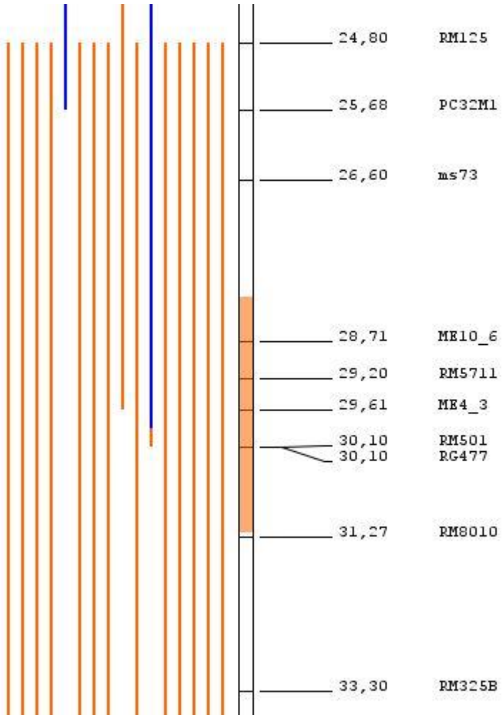

The orange region represents MQTL7.3 which has the maximum number of QTL associated with drought tolerance in rice. The genetic distance of this MQTL is a 2.67cM and flanked by ME10_6 and RM8010

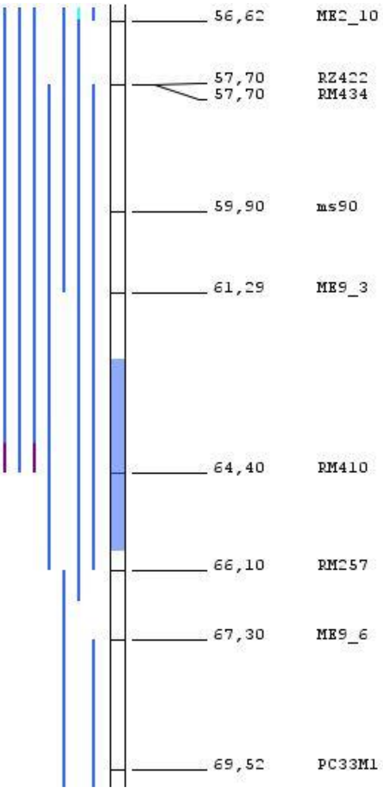

The blue region represents MQTL 9.7 which has the maximum number of QTL associated with drought tolerance in rice. The genetic distance of this MQTL is a 2.87cM and flanked by ME9_3 and RM257
